# Study of the factors influencing load displacement curve of energy absorbing device by area division simulation

**DOI:** 10.1038/s41598-022-17784-x

**Published:** 2022-08-05

**Authors:** Dong An, Tianwang Liu, Hewei Cui, Zheng Chen, Hailiang Xu, Yimin Song

**Affiliations:** School of Civil Engineering, North China University of Technology, Beijing, China

**Keywords:** Civil engineering, Devices for energy harvesting

## Abstract

A pre folded energy absorbing device, which is the key device of energy absorption anti impact for roadway support, is tested by quasi-static compression and simulated. The energy absorbing device is divided into zones, and the influence of the area on the load displacement curve of the energy absorbing device is studied according to the area. It is found that the error of numerical simulation is within 5%, indicating that the finite element modeling procedure is appropriate for the problem analyzed here. The device crushes following the pre folded origami pattern in a stable progressive. The device was divided into four areas: the upper and lower opening region of the concave surface four corner parts; the other areas of opening regions; the middle fold edge; the surrounding four sides edge. Each area has effect on the first drop stage and the second rise stage of the load displacement curve. The middle fold edge area has an effect on the peak load value of load displacement curve. Four indicators of peak load, average load, load efficiency, and specific energy absorption were generated from the results of numerical simulation. The strength enhancement of corner region can ensure the energy absorbing device with low peak load and high mean crushing load. The other areas of opening regions affect the first descending and second ascending of the curve. The first rising stage bears the load from the middle edge.

## Introduction

With mining of coal resources towards depth, rock burst accidents occur frequently, which has affected the safety of working face and roadway. Rock burst in coal mine refers to the dynamic phenomenon that the rock around the wellbore or working face suddenly occurs severe damage due to the instantaneous release of elastic deformation energy, which is often accompanied by the drop of coal and rock, huge noise and gas waves. It is destructive and one of the major disasters in coal mines. The effective prevention and control technology of rock burst is the support method^1^. It is a passive protection method to improve the ability of supporting body to resist rock burst by increasing the support strength or improving the support method. The working face has support, and the roadway also needs support. Roadway support includes a variety of types. Lv et al.^2,3^ established a mechanical model of rigid—flexible energy absorbing support structure. Cheng et al.^4^ elaborates its effective anti-surge mechanism against impact loads by means of bracket bearing deformation characteristics. Zhang et al.^5^ proposed anchor rod (cable) and U-shaped frame support failure types and control technology. Zhang et al.^6–8^ verifies the effectiveness of applying portal brackets to solve practical engineering problems in actual projects. Fan et al.^9^ proposed three pressure frame warning indicators based on stent position identification. Chen et al.^10^ designed and developed a self-moving flexible shield hydraulic support, which was successfully applied in the mining of sharply inclined coal seam. Zhang et al.^11^ analyzes the mechanical performance of two-prop shield caving hydraulic support. Tian, Q.^12^ developed a support shield type hydraulic support to provide a technical reference to prevent the hydraulic support from tipping and sliding in large inclination coal seam mining. Hydraulic column support is an important means, energy absorption anti-impact support^13–15^ is an effective form, which can achieve the support effect by yielding and giving way.

Energy absorbing device is a key part of the support in the energy absorption support system. It can release the impact of the surrounding rock by rapid yield and protect the support system from damage^16^. There have been many studies on energy absorption devices in other fields. In the field of traffic, such as vehicle crash prevention, support role in the process of aircraft cargo hold crash, and crash prevention role in modern rail vehicle collision^17–21^. The energy absorbing devices appear as a progressive fold in axial compression, and the plastic deformation of the fold can absorb a large amount of energy^22^. In recent years, many experts and scholars have optimized the design of the energy-absorbing capacity of the energy absorbing devices, Wang et al.^23^ improves the energy absorption capacity by optimizing the cross-sectional geometry of the energy absorption device. Tarlochan et al.^24^ selects thin-walled structures with cross-sectional shapes that meet performance requirements to improve crash performance. A, A. Nia. et al.^25^ found that circular tube has the most energy absorption capacity and the most average force among all investigated sections by studying thin-walled tubes with different cross-sectional shapes. Zarei ^26^ applied multidesign optimization technology to optimize honeycomb filled of aluminum to maximize the absorption of energy and specific energy. Yalcin ^27^ showed through experiments that the proper PVC foam-filled circular aluminum tube has a significant effect on energy absorption capacity. Xing et al.^28^ analyzed the axial energy absorption characteristics of aluminum honeycomb buffers through engineering examples and numerical simulations. Zhang et al.^29^ discusses the relationship between shape parameters of honeycomb cell and dynamic performance of isolator. Yuan et al.^30^ can effectively improve the energy absorption capacity of the composite structure by optimizing the material composition and structure design. In the mining field, there are many studies on supports, but the research on pre folded energy absorbing device is not enough.

The working mechanism of the energy absorbing device is that the energy absorbing device converts the impact energy in the collision process into plastic deformation energy by its own buckling, fracture and other failure forms. The larger the plastic deformation area of the structure, the more the energy absorbed and converted in the deformation process. The folding process and mechanical properties of a pre folded energy absorbing device were studied by quasi-static compression test and simulated by ABAQUS, the characteristics and causes of the load displacement curve of the energy absorbing device were analyzed, the impact process determines that the support should be given up first and then resisted. The best support curve should be constant resistance. The energy absorption curve of the support has the largest energy absorption and the strongest resistance. Most of the existing energy absorption curves are W-shaped. Therefore, it is necessary to study the energy absorbing device, improve the support effect, improve the bearing capacity of columns, improve the application level of energy absorption components and improve the support capacity of energy absorption support.

Based on the numerical simulation of the quasi-static compression test on the energy absorbing device, this paper studies the characteristics of the load displacement curve of the energy absorbing device. On the premise that the energy absorbing device is based on the strain zoning, this paper compares the parameters of the energy absorbing device after strengthening in different areas, and finally puts forward the optimization objectives and engineering practices for the energy absorbing device.

### Energy-absorbing device

#### Energy absorption anti-support application

**Figure 1:**
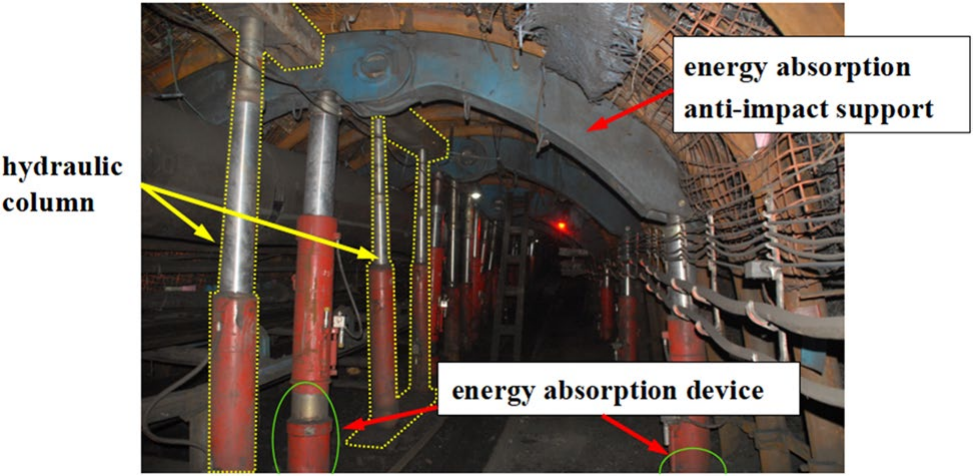
Photograph of energy absorption anti-impact support in the roadway.

**Figure 2:**
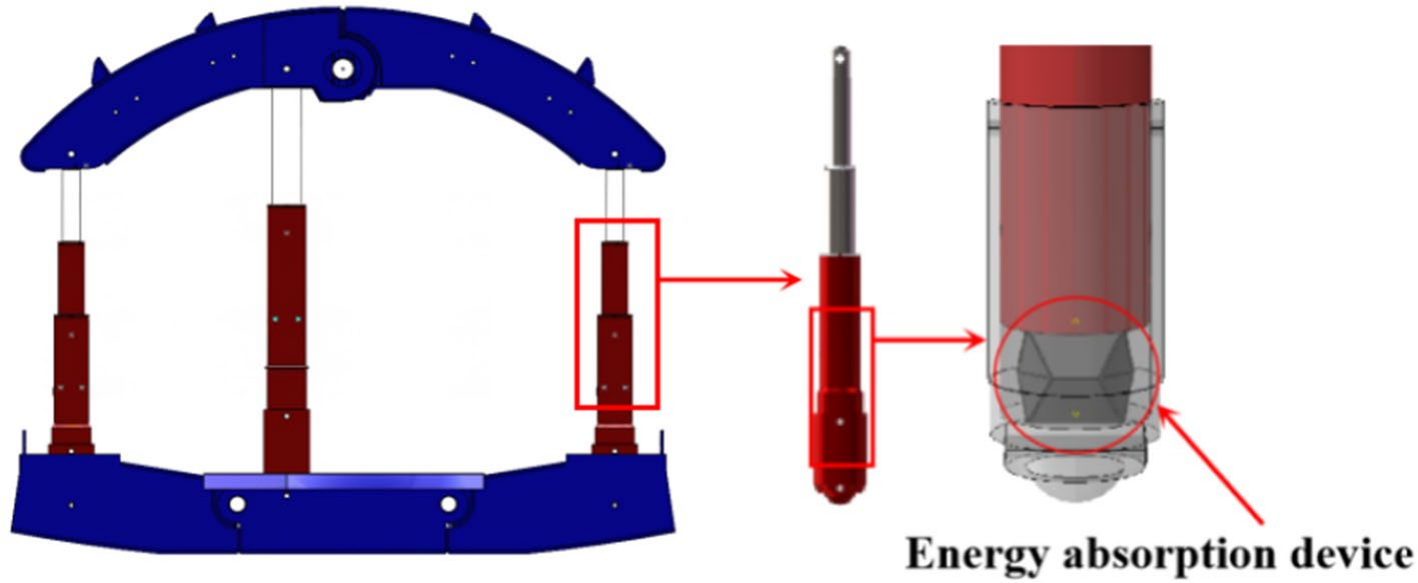
Location of the energy absorbing device in the structure.

This case of energy absorbing device in coal mine is located in Henan Province, China. The coal mine roadway is supported by anchor mesh, anchor rods, anchor cables, hydraulic lifting sheds, and hydraulic support for rock burst prevention^31^. The hydraulic support in the coal mine roadway adopts energy absorption anti-impact device, which is shown in Fig. 1. Its main structure consists of four parts: arched top beam, micro arc base, hydraulic column and anti-impact device, forming a symmetrical arched frame structure, which is shown in Fig. 2. The arched top beam is mainly used to support the surrounding rock at the top of the roadway. Three hydraulic columns are supported between the top and bottom beams, providing working resistance for the support. The energy absorbing device provides working resistance together with the column during quasi-static support and can quickly deform and absorb energy in case of sudden large surrounding rock impact, so as to realize a deformation process of the whole support.

#### Dimensions

**Figure 3:**
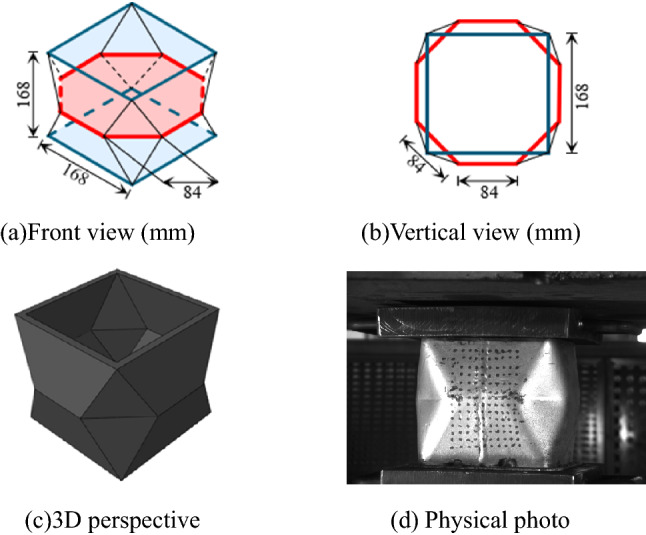
Dimensions of the pre folded energy-absorbing device.

The energy absorbing device uses the origami pattern^32^. The dimension of the device is shown in Fig. 3. The change of the inclined folds angle corresponds to the shape of the wall plate and the height of the cylinder. Since the deformation of the pre folded square is a fixed pattern and predictable, it can be applied as a parameterizable energy absorbing device by studying the relationship between its geometric relationship, material properties, and the buckling characteristics of the pre folded square structure.

#### Materials and production method

**Figure 4:**
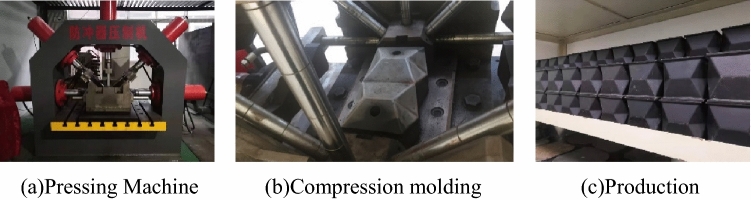
Production of pre folded square tube for energy absorbing device.

Theoretically, gradually folding a flat sheet along the creases and then joining the two opposite free edges, a square origami pre folded box shown in Fig. 3(a) can be constructed^33^. In fact, the energy absorbing device is made of Q690^34^, one of the high strength steel plates in China^34^. The steel plate is 10 mm in thickness and cannot be folded and enclosed by a whole steel plate. The energy absorbing device is processed by butt welding two half shells as a whole. The specific steps are as follows: (1) Steel plate cutting; (2) Half shell bending; (3) Edge trimming; (4) Butt welding; (5) Heat treatment. The half shell of the pre folded square tube specimen is folded and pressed by a group of molds and their supporting press, as shown in Fig. 4. In order to prevent excessive damage at the bending position, the steel plate needs to be preheated before bending. The formed pre folded square tube specimen is quenched first, and then tempered to eliminate the residual stress at the bend of the steel plate and near the weld. Such process inevitably affects the mechanical properties of the energy absorbing device, thus affecting the anti-impact performance of the road way support.

### Quasi-static compression test and numerical simulation

#### Quasi-static test

Quasi-static compression test was conducted on the energy absorbing device to obtain the load displacement curve and characteristic of energy absorption. The hydraulic servo-controlled testing machine with model number RLJW-2000 was used for loading by means of displacement loading. The dynamic loading range was 3000 kN and the maximum loading rate was 8 m/s. The whole deformation process was observed by high-definition photograph. At the same time, the displacement deformation and impact pressure were measured.

#### Numerical simulation

The research object of this paper is the pre folded energy absorbing device made of high-strength steel, which has large deformation, so the finite element analysis software package ABAQUS/Explicit^35^ was applied to simulate the axial compression process. The compression test was modeled as the energy absorbing device standing on a fixed stationary rigid panel and being compressed by a moving one with the test loading rate. Downward displacement was assigned to the moving rigid panel to control the compressing process to free degree, and smooth amplitude definition built in ABAQUS was assigned to the control the loading rate. The final distance of compression simulate was 120 mm. Four-node shell elements with reduced integration S4R were used to mesh the device. Self-contact was employed to model the contacts among the device itself. The upper and lower edges of the device were in a frictional contact relationship to the rigid panels considering the friction coefficient taken as 0.3. Density as 7650 kg/m^3^, Young’s Modulus as 210 GPa, Yield strength as 690 MPa, the thickness of the energy absorption device as 6 mm, the step is only one and dynamic explicit, and the time period as 0.02 s, nlgeom is on, time scaling factor as 1, frequency of field output is evenly spaced time intervals, the interval as 200, Ultimate strain as 0.2, Poisson’s ratio as 0.3 were used to describe the strength/strain criterion of the material, and the dimensions of the device was consistent with the test specimen.

**Figure 5:**
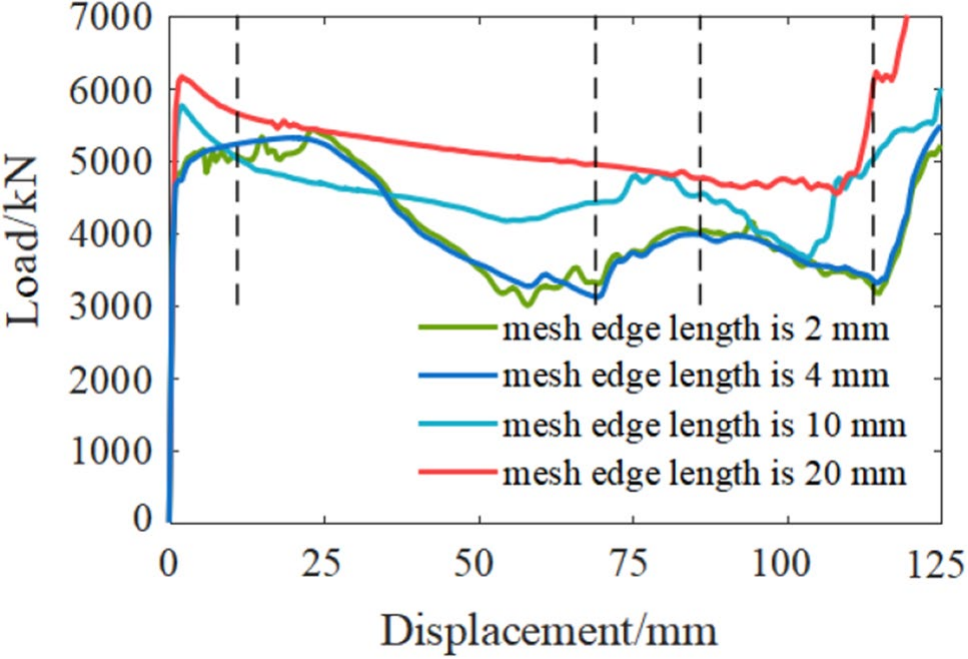
Simulation results of different mesh size.

**Figure 6:**
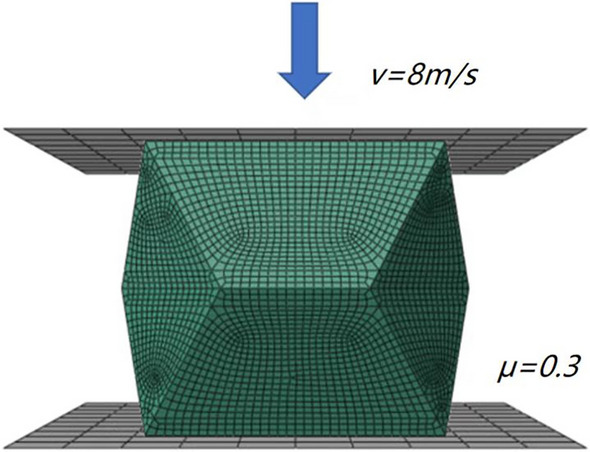
Numerical simulation model.

The accuracy of the ABAQUS calculation is affected by the mesh^36^, as shown in Fig. 5, the load displacement curves were totally different depending on the mesh. The global mesh size of 4 mm and analysis time of 0.02 s were chosen to simulate. The device model was divided into 7536 grid cells. The numerical model is shown in Fig. 6.

#### Test and simulation curves and validation

**Figure 7:**
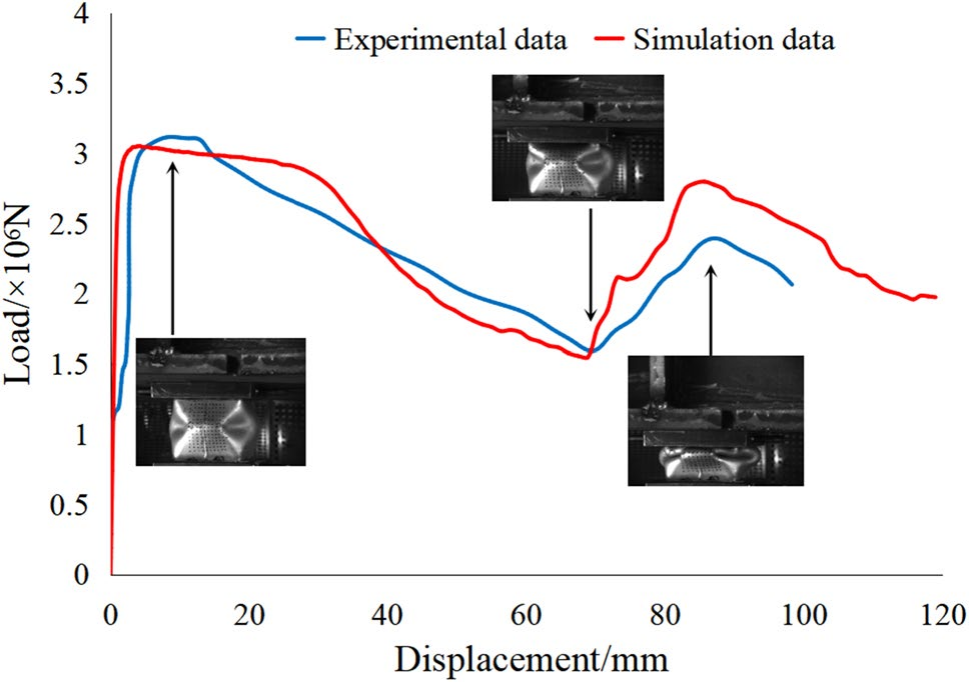
The load displacement curve of test and simulation.

The load displacement curve of test and simulation, with the compression process in the test is shown in Fig. 7, which is of W shaped with up and down fluctuation as said above. From the numerical value in Fig. 7, yield load (*F*_max_) in test was 3115.29 kN, approximately equal to that in simulation which value is 3020.51 kN. Minimum load capacity during deformation (*F*_min_) in test was 1604.22 kN, approximately equal to that in simulation which value is 1529.77 kN. The error of numerical simulation is within 5%, indicating that the finite element modeling procedure is appropriate for the problem analyzed here.

#### Discussion

Although the load displacement curves obtained from compress test and numerical simulation were approximately the same, in fact, the material properties were changed due to bending and overheating during production process, which leads to impossible to accurately simulate the load curve of the energy absorption device. Due to reference^37^, the strain of the energy absorbing device in the crushing process varies according to different region. It shows that the production process of the energy absorbing device affects the material properties of each position.

### Influence to load displacement curve

#### Crush mode and plastic hinge

**Figure 8:**
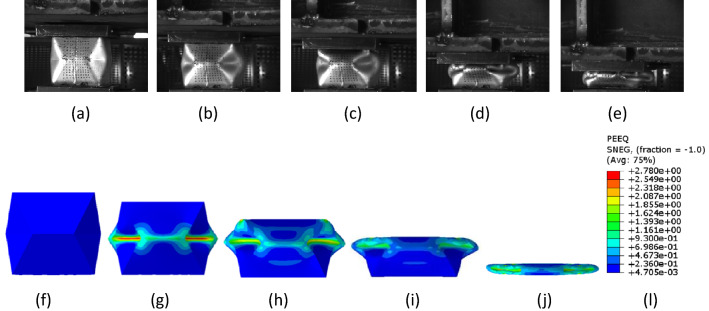
Crush process and PEEQ contour map.

Figure 8 shows the compression process of the device, and the corresponding PEEQ contour maps are plotted. It can be seen that the device crushes following the pre folded origami pattern in a stable progressive. At the beginning, the middle pre folded zone first starts to fold, meanwhile the load raised rapidly. From the PEEQ, the second map indicates that two pairs of traveling plastic hinge lines are formed along the four sides, the load rose to peak. As the device was further compressed, the traveling plastic hinge lines moved away from each other, deforming the corner areas, as can be observed in the third map, load began to drop. The upper half of the device collapsed, and the folding area generated stack deformation. After the upper was totally folded, the lower began to collapse until the whole device was completely crushed. It is proved that the device has a stable and predictable deformation process. It can be seen from the PEEQ contour maps in Fig. 8 is that plastic deformation was limited to the zones, such as the edge, the fold line, the corner, whereas the remaining panels undergo small plastic deformation. Therefore, when analyzing the load displacement curve of energy absorbing device, it should be divided into different areas.

#### Area division

**Figure 9:**
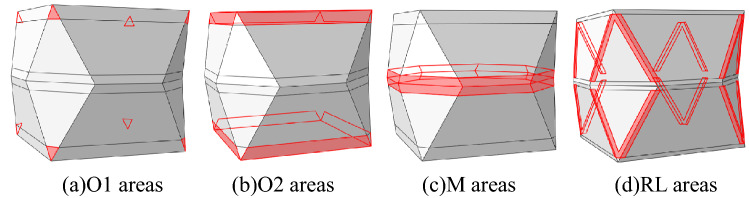
Model partition schematic.

According to the characteristics of the compression deformation and the plastic zones, the energy absorbing device was divided into several different areas. It was divided into four areas: the upper and lower opening region of the concave surface four corner parts is named O1; the other areas of opening regions named O2; the middle fold edge named M; the surrounding four sides edge named RL, which is shown in Fig. 9.

#### Work conditions

The load displacement curve of energy absorbing device was affected by the evolution of plastic zone, and the range of different areas was calculated according to the following Table1. The unchanged original device was named IM for comparation.

**Table 1:** Assumed influenced dimension of each area.

Work conditions	Model	O1(mm)	O2(mm)	M(mm)	RL(mm)
1	IM	0	0	0	0
2	O1-10	10	0	0	0
3	O1-15	15	0	0	0
4	O1-O2-10	10	10	0	0
5	O1-O2-15	15	15	0	0
6	M-10	0	0	10	0
7	O1-O2-M-10	10	10	10	0
8	O1-O2-M-RL-10	10	10	10	10

#### Evaluation Indicator and results

Based on the performance requirements of different areas, four indicators of peak load, average load, load efficiency, and specific energy absorption^38^ were generated from the results of numerical simulation, the following indicators are described:

(1) Peak load

1 $$F_{max} = \max [F(s)]$$

In Eq. (1), *F*(s) is the time history of the load in the compression process. The peak load *F*_max_ is the threshold value when the energy absorbing device starts to crush.

(2) Average load

2 $$F_{mean} = \frac{{\smallint F({\text{s}}){\text{d}}s}}{S}$$

In Eq. (2), *S* is the total compression displacement of the energy absorbing device. *F*_mean_ is the energy absorption per unit compression displacement, which reflects the overall energy absorption capacity.

(3) Load efficiency

3 $$F_{{\text{E}}} = \frac{{F_{mean} }}{{F_{\max } }}$$

In Eq. (3), the load efficiency is the ratio of the average load to the peak load, *F*_E_ ∈ (0,1). The smaller the value, the stronger the volatility of the load during compression, and the closer it is to 1, the more stable it is.

(4) Specific energy absorption

4 $$SEA = \frac{E}{m}$$

In Eq. (4), m is the total mass of the energy absorbing device, and E is the total energy absorption of the device, which is calculated by the Eq. (5):

5 $$E = \smallint F(s){\text{d}}s$$

The evaluation indicators results are shown in Table 2.

**Table 2:** Evaluation Indicators.

Work conditions	Number	*F*_max_(kN)	*F*_mean_(kN)	*F*_E_	*SEA*(kJ/kg)
1	IM	3049.64	1940.46	0.64	31.84
2	O1-10	3056.12	2556.93	0.84	41.96
3	O1-15	3046.42	2514.92	0.83	41.27
4	O1-O2-10	3062.13	2576.31	0.84	42.28
5	O1-O2-15	3048.15	2501.49	0.82	41.05
6	M-10	3214.36	2504.54	0.78	41.10
7	O1-O2-M-10	3059.49	2605.51	0.85	42.76
8	O1-O2-M-RL-10	3046.42	2514.92	0.83	41.27

**Figure 10:**
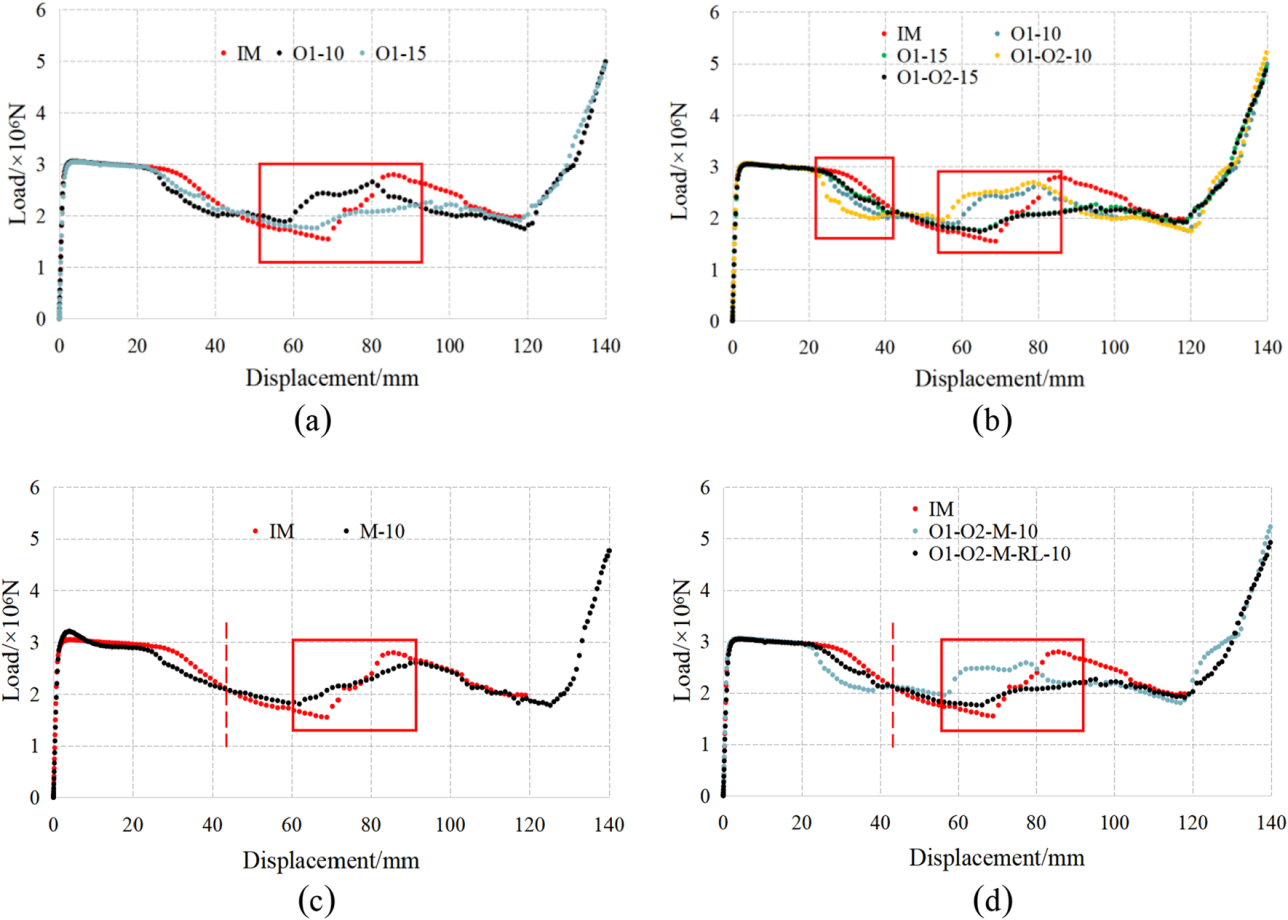
Load displacement curves.

According to the numerical simulation results, the load displacement curves of the energy absorption under work conditions are shown in Fig. 10.

#### Effects of corner region (O1)

The numerical data in Table 2 show that F_max_ of O1-10 and O1-15 is 3056.12 kN and 3046.42 kN separately, was approximately equal to that of IM. The range of O1 area basically does not affect the peak load of the device. F_mean_ of O1-10 is 2556.93 kN, 31.77% and higher than that of IM, whereas F_E_ of O1-10 is 0.84, 31.25% higher than that of IM. O1-15 has almost the same numerical data. That means the strength enhancement of corner region can ensure the energy absorbing device with low peak load and high mean crushing load. Besides, SEA of O1-10 and O1-15 is significantly improved, which is 31.78% and 29.62%, higher than that of IM, respectively.

It can be seen from Fig. 10a that there is basically no difference of the load value before the compression displacement is 20 mm, no matter the O1 area changes. The first decline stage of O1-10 and O1-15 is significantly earlier than that of IM. The load began to rise again when the compression displacement is 60 mm for O1-10 and 65 mm for O1-15, earlier than 70 mm for IM. That means the minimum value of O1-10 and O1-15 is higher than that of IM. Therefore, it can be concluded that the load displacement curve is stable. With the increase in O1 width, the first decline section and the second rise section of the load displacement curve tend to be flat, indicating that the O1 region affects that of the curve.

#### Effects of opening region (O2)

Keep O1 area changed, the numerical data in Table 2 show that F_max_ of O1-O2-10 and O1-O2-15 is 3062.13 kN and 3048.15 kN separately, was also approximately equal to that of IM. The O2 region does not affect the fluctuation of the peak load. The range of O2 area basically does not affect the peak load of the device. F_mean_ of O1-O2-10 is 2576.31 kN, 32.77% and higher than that of IM, whereas F_E_ of O1-O2-10 is 0.84, 31.25% higher than that of IM. O1-O2-15 has almost the same numerical data. That means the strength enhancement of opening and corner region can ensure the energy absorbing device with low peak load and high mean crushing load. Besides, SEA of O1-O2-10 and O1-O2-15 is significantly improved, which is 32.79% and 28.93%, higher than that of IM, respectively. Under the influence of O1 region, O2 region has little effect on the curve.

It can be seen from Fig. 10b that there is basically no difference of the load value before the compression displacement is 20 mm, no matter the O2 region changes. When the O1 width is 10 mm, when the displacement is between 20 and 40 mm, changing the O2 width caused the curve decreased. When the displacement is between 50 and 80 mm, the curve increased. However, when the O1 width is 15 mm, the curve basically does not change after O2 changed. This shows that the O2 region affects the first descending and second ascending of the curve.

#### Effects of middle edge (M)

The numerical data in Table 2 show that F_max_ of M-10 is 3214.36 kN, which is 5.4% higher than that of IM, indicating that M region has a certain influence on the peak load. The peak load of the device is obviously improved by strength enhancement of middle edge. F_max_ of O1-O2-M-10 is 3059.49 kN, which is 0.32% higher than that of M-10. F_mean_ of M-10 is 2504.54 kN, 29.07% higher than that of IM, 3.88% lower than that of O1-O2-M-10. Simultaneously changed the O1 and O2 region, the peak load will be decreased, the mean load will be improved. Whereas F_E_ of M-10 is 0.78, 21.88% higher than that of IM. That means the strength enhancement of middle edge region can ensure the energy absorbing device with both high peak load and high mean crushing load. Besides, SEA of M-10 is significantly improved, which is 29.08%, higher than that of IM.

It can be seen from Fig. 10c that compared with the curve of IM; the initial stage is improved by the change of M region. In the first decline stage, the curve is gentle and the valley bottom is obviously advanced and improved. The curve is lower than the initial model curve before the displacement of 40 mm. After the displacement of 40 mm, the curve is higher than that of IM. In the second rising stage, the peak value of the curve obviously delayed and decreased and the curve was flat. It shows that the first rising stage bears the load from the middle edge.

#### Effects of side edge (RL)

The numerical data in Table 2 show that F_max_ of O1-O2-M-RL-10 is 3046.42 kN, was also approximately equal to that of IM. F_mean_ of O1-O2-M-RL-10 is 2514.92 kN, 29.60% and higher than that of IM, whereas F_E_ of O1-O2-M-RL-10 is 0.83, 29.69% higher than that of IM. That means the strength enhancement of middle edge region can ensure the energy absorbing device with both high peak load and high mean crushing load. Besides, SEA of O1-O2-M-RL-10 is significantly improved, which is 29.62%, higher than that of IM.

It can be seen from Fig. 10d that there is a significant change in the second half of the curve. The second peak load of ‘W’ type hardly appeared. The first half load does not change when the RL region changed. The first decline and the second rise of the curve were more stable, and the first decline stage decreased significantly and was delayed about 10 mm. When the displacement is between 20 and 40 mm, the curve is obviously improved, which is between 50 and 60 mm, the load is obviously reduced about 1000 kN.

## Conclusion

A pre folded energy absorbing device was tested by quasi-static compression and simulated. Numerical simulation results show the finite element modeling procedure is appropriate. According to the characteristics of the compression deformation and the plastic zones, the energy absorbing device was divided into several different areas The influence of the area on the load displacement curve of the energy absorbing device is studied. Four indicators of peak load, average load, load efficiency, and specific energy absorption which were generated from the results of numerical simulation were used to the effects.

Each area has effect on the first drop stage and the second rise stage of the force–displacement curve. The middle fold edge area has an effect on the peak load value of force–displacement curve. The strength enhancement of corner region can ensure the energy absorbing device with low peak load and high mean crushing load. The other areas of opening regions affect the first descending and second ascending of the curve. The first rising stage bears the load from the middle edge.

Fmax of M-10 is 3214.36 kN, which is 5.4% higher than that of IM. Fmax of O1-O2-10, O1-O2-15, O1-O2-M-10, O1-O2-M-RL-10 were also approximately equal to that of IM. Fmean of O1-O2-10, M-10, O1-O2-M-RL-10 is 32.77%, 29.07% 29.60% higher than that of IM. SEA of O1-O2-10, O1-O2-15, M-10, O1-O2-M-RL-10 is significantly improved, higher than that of IM.

From a comprehensive view of the four aspects of peak load, average load, load efficiency and specific energy absorption, O1-O2-M-10 can play a good role, and its average load, load efficiency and specific energy absorption are improved most, which are 32.77%, 32.81% and 34.30% higher than IM, respectively. It has the best effect on improving the energy absorption curve of energy absorption device.

The process of impact determines that the support should give way first and then resist. The best support curve should be constant resistance, so that the energy absorption curve of the support has the largest energy absorption and the strongest resistance. However, most of the existing energy absorption curves are W-shaped. Therefore, The research on the pre-folding energy absorption device can improve the energy absorption curve of the support, improve the support effect, improve the bearing capacity of the column, improve the application level of energy absorption components and improve the support capacity of energy absorption support, so as to strengthen the support ability of the roadway and reduce the harm caused by the rock burst.

## Supplementary Information

Supplementary Information.

## Data Availability

All data, models, or code that support the findings of this study are available from the corresponding author upon reasonable request.
